# A Systematic Review of Potential Therapeutic Use of Lycium Barbarum Polysaccharides in Disease

**DOI:** 10.1155/2019/4615745

**Published:** 2019-02-12

**Authors:** Sum Sum Kwok, Yashan Bu, Amy Cheuk-Yin Lo, Tommy Chung-Yan Chan, Kwok Fai So, Jimmy Shiu-Ming Lai, Kendrick Co Shih

**Affiliations:** ^1^Department of Ophthalmology, Li Ka Shing Faculty of Medicine, University of Hong Kong, Hong Kong; ^2^Department of Ophthalmology, Hong Kong Sanatorium and Hospital, Hong Kong

## Abstract

**Objective:**

To evaluate the effect of* Lycium barbarum* polysaccharides in the treatment and/or prevention of diseases of different etiologies and systems.

**Methods:**

We performed an Entrez PubMed literature search using keywords “lycium”, “barbarum”, “polysaccharides”, “anti-fibrotic”, “anti-apoptotic”, “anti-oxidizing”, “anti-aging”, “neuroprotection”, “metabolism”, “diabetes”, “hyperlipidemia”, “neuroprotection”, and “immunomodulation” on the 14^th^ of August 2018, resulting in 207 papers, of which 20 were chosen after filtering for ‘English language' and ‘published within 10 years' as well as curation for relevance by the authors.

**Results:**

The 20 selected papers included 2 randomized control trials (1 double-blinded RCT and 1 double-blinded placebo-controlled RCT), 11 in vivo studies, 5 in vitro studies, 1 study with both in vivo and in vitro results, and 1 chemical study. There is good evidence from existing studies on the antifibrotic, antioxidizing, neuroprotective, anticancer, and anti-inflammatory effects of* Lycium barbarum* polysaccharides. However, there is a need for further studies in the form of large-scale clinical trials to support its use in humans. There is also significant potential for LBP as a safe and effective topical treatment in ocular surface diseases, owing to promising in vitro results and a lack of demonstrated toxic effects to corneal epithelial cells.

**Conclusion:**

Results from existing studies suggest that LBP is a promising therapeutic agent, particularly in the management of liver disease, hyperlipidemia, and diabetes. One major limitation of current research is a lack of standardization and quality control for the LBP used. The availability of research-grade LBP will inevitably promote future research in this field worldwide.

## 1. Introduction

Wolfberries or* Lycium barbarum* have been used in Traditional Chinese Medicine (TCM) or as a cooking ingredient for over a thousand years [1]. Its use in TCM is credited to its abilities to balance the “yin” and “yang” within the body as well as nourishing the eyes, kidney, lungs, and liver [2, 3]. They grow naturally within the north western Chinese provinces particularly in Ningxia.

The main constituents of wolfberries include* Lycium barbarum* polysaccharides (LBP), scopoletin, and 2-O-*β*-D-glucopyranosyl-L-ascorbic acid (AA-2*β*G) [4]. We will be focusing on the therapeutic effects of LBP; the key bioactive ingredient in wolfberries [5].* LBP* consist of 6 monosaccharides, namely, glucose, arabinose, galactose, mannose, rhamnose, and xylose [6]. They are popular health supplements, noted as being “superfood,” owing to their s wide range of purported health benefits.

From published studies, LBP intake is associated with a number of therapeutic effects, including antiaging, [7] owing to their antioxidizing properties, metabolic effects [8], neuroprotective effects in neurodegeneration [9] and neurotoxicity [10], and ocular neuroprotective effects [11]. Furthermore, there is evidence that LBP may even improve male fertility [12]. Additionally, LBP has been demonstrated to have an excellent safety profile, with no documented adverse reactions related to its usage. However, due to its origins as a health supplement, there is a lack of awareness amongst the medical and scientific community regarding published work on the use of LBP as a therapeutic agent, while there remain a number of significant issues that need to be addressed; chiefly because of the lack of proven consistency in the quality and source of LBP product used for the different published studies, we believe a review of current evidence will increase awareness in this promising area of research and promote large-scale clinical trials in this field.

In this review article, we will systematically examine the available evidence from published studies on the therapeutic potential of LBP supplementation in health and disease for different functional systems of the human body.

## 2. Methods

**Figure 1:**
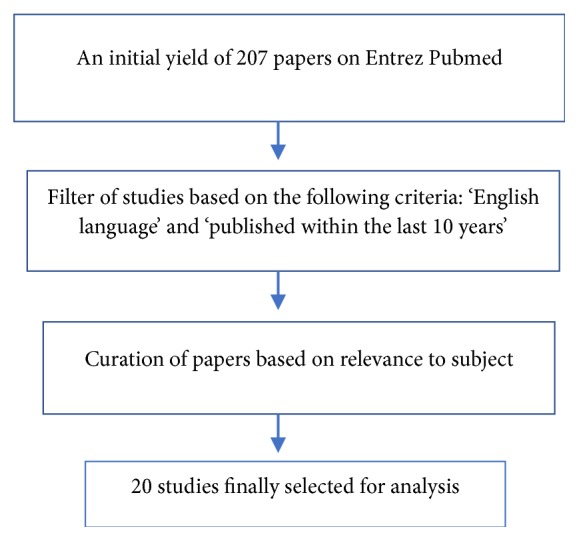
Flowchart of search strategy.

An Entrez PubMed search was performed on August 28^th^, 2018, using keywords “lycium”, “barbarum”, “polysaccharide”, “anti-fibrotic”, “anti-apoptotic”, “anti-oxidizing”, “anti-aging”, “metabolism”, “diabetes”, “hyperlipidemia”, “neuroprotection”, and “immunomodulation” which yielded 207 publications. Filters were set to select original articles published in the last 10 years which are available in full text and written in English, resulting in 154 papers. The resulting papers were then reviewed by SSK and KCS for subject relevance via abstract or the full text of the article. For example, papers that either investigated wolfberry extract rather than LBP or investigated mixed polysaccharides that included LBP were excluded from analysis. This was done in order to avoid misattributing health benefits derived from other components to LBP. Finally, 20 papers were selected and analyzed for this study (see Figure 1). The papers were stratified based on their study designs as well their potential therapeutic effects, including antiaging effects, neuroprotective effects, anticancer effects, and immunomodulatory effects.

## 3. Results

Amongst the 20 publications selected, only 2 were clinical studies, with both being double-blinded placebo-controlled randomized control trials. Of the remaining 18 laboratory-based studies, 11 were in vivo studies, 5 were in vitro studies, and 1 study had both in vivo and in vitro results. We summarized the results of the 20 studies in three separate tables using the following subject headings: (1) clinical trials on LBP, (2) translational studies on systemic therapeutic effects of LBP, and (3) translational studies on local therapeutic effects of LBP (see Tables 1, 2, and 3). As the studies were diverse in their study designs and outcomes, the results could not be combined for meta-analysis. Overall, from existing data, there is an evident lack of well-designed randomized control trials to evaluate the effect of LBP in humans. Though there are published RCTs for investigating its effect in diabetes and diabetic retinopathy, the respective sample size of the studies is insufficient for the results to have significant impact in clinical management. Nevertheless, the promising findings identified from both clinical studies and translational work should warrant greater interest in this field of research.

### 3.1. Systematic Effects

#### 3.1.1. Effects on Metabolism

Cai et al. published a double-blinded, placebo-controlled RCT on 67 patients with type 2 diabetes mellitus, comparing blood glucose and lipid levels of patients given 300mg/day LBP treatment perorally compared to type 2 diabetic controls. It was found at 3 months that patients on LBP treatment had significant increase in high-density lipoproteins (HDL) levels from baseline compared to controls. Moreover, the areas under curve (AUC) for blood glucose levels decreased significantly compared to the control during the study period (-7.86% vs 1.61%, respectively). Furthermore, the effect of LBP on glucose control was more significant in subjects who were not taking any oral hypoglycemic agents [13]. Thus, LBP is a potential dietary supplement for those with diabetes, particularly for newly diagnosed cases where dietary control is started as initial management. However, given the small sample size of this study, a large-scale multicenter RCT is needed to verify LBP's hypoglycemic effect in humans.

Wang et al. evaluated the effect of LBP on streptozotocin-induced diabetic male Sprague-Dawley rats by comparing treatment with 0.5 ml 6% LBP solution delivered intragastrically for 4 weeks compared to 0.5% normal saline delivered in the same way for 4 weeks. There was significant reduction in vascular endothelial growth factor (VEGF) mRNA levels of the retina in the LBP treatment group compared to diabetic controls. Transmission electron microscopy of the retinal ganglion cells and photoreceptors exhibited marked differences between groups. LBP treated diabetic rats had no obvious evidence of cellular damage, while the retina of controls showed evidence of fractured mitochondrial cristae and round vacuoles within the mitochondria. VEGF is an important factor in diabetic retinopathy disease-progression, as VEGF increases permeability of the blood-retinal barrier and promotes retinal neovascularization. The results from this study demonstrated the ability of LBP to improve blood glucose control in an experimental model of insulin-dependent diabetes mellitus and the associated risk of microvascular complications. Additionally it appears to have a direct effect on attenuating experimental diabetic retinopathy disease progression [14]. However, it is important to note that although LBP demonstrates antioxidizing and antineovascular effects in the experimental setting, whether it can improve clinical outcomes is questionable, as it is unknown whether the observed biochemical and histological changes are sufficient to prevent or slow the progression of diabetic retinopathy in the real-world setting.

In a study by Pai et al., the lipid lowering effect of LBP was compared with conventional treatment in rats exposed to a cholesterol-rich high fat diet for 45 days. Briefly, Wistar albino rats were fed* Lycium barbarum *(LB) fruit extract (250mg/kg or 500mg/kg) for 30 days following the cholesterol-rich diet. The effect of LB was compared to atorvastatin (10mg/kg/day), a commercially available statin-group lipid-lowering drug. It was found that LB treatment, at either dosages, resulted in significant reduction in the total blood cholesterol, triglycerides, and very low-density lipoprotein-cholesterol (VLDL) levels. Furthermore, for the 500 mg/kg LBP-treated group, there was a significant reduction of blood low-density lipoproteins (LDL) compared to controls, while there was a significant elevation in blood HDL in the 250mg/kg LBP-treated group. In comparison, the atorvastatin-treated controls only demonstrated significant reduction in total blood cholesterol levels, with no observable effect on blood triglycerides and lipoproteins [15]. In comparison, it was demonstrated that LBP at 500mg/kg reduced triglyceride and VLDL level to a significantly greater extent than atorvastatin treated rats. This promising observed effect should be further assessed in human clinical trials.

A double-blind, placebo-controlled randomized control trial in humans by Amagase et al. investigated the effect of LBP intake on basal metabolic rate. The paper detailed two separate studies assessing the effect of* Lycium barbarum* fruit juice (30/60/120ml doses) on the resting metabolic rate (breath oxygen volume VO2) and postprandial energy expenditure in 8 subjects and on the waist circumference of 33 subjects. Compared to the placebo-treated subjects,* Lycium barbarum* juice-treated subjects had increased VO2 1-4 hours after ingestion in a dose-dependent manner. At 1 hour after ingestion the VO2 in the 120ml* Lycium barbarum* juice-treated group increased significantly by 58.26 ± 5.72 ml/min compared to baseline and as well as compared to the placebo-treated group [16]. As for waist circumference of subjects after 14 days of intervention, the LB juice-treated group showed a significant reduction in waist circumference by 5.54 ± 0.65cm compared to baseline. This paper suggests that LBP has potential in regulating basal metabolism in humans. It is worth noting however that the sample size for this RCT was small, without evidence of power calculation during the study planning period.

In another study the effect of LBP was assessed in hyperlipidemic mice subject to chronic composite psychological stress. [17]. Briefly, Kun Ming (KM) mice were divided into controls, hyperlipidemia alone (H), hyperlipidemia + LBP treatment (HL), hyperlipidemia + chronic composite psychological stress (HS), and finally hyperlipidemia + LBP treatment + chronic composite psychological stress group (HLS) [17]. For experiments LBP was given at 80 mg/kg/day for 30 days. A high cholesterol diet was used for induction of hyperlipidemia. The groups were compared for hepatic malondialdehyde (MDA) levels; a proxy for liver damage, serum heat-shock protein 70 (HSP-70) levels; a marker for oxidative stress and serum interleukin 6 (IL-6) levels; a proinflammatory and profibrotic cytokine. The results showed that while MDA, HSP-70, and IL-6 levels were significantly elevated in hyperlipidemic groups compared to controls, the inclusion of LBP treatment significantly lowered these markers in HLS and HL groups. This demonstrates the potential role for LBP in ameliorating high cholesterol and stress-induced hepatic and vascular damage. Furthermore, LBP treatment was shown to significantly increase liver mRNA levels of CYP7A1 in HL and HLS groups compared to controls as well as H and HS groups. CYP7A1 is a rate-limiting enzyme involved in the hepatic conversion of cholesterol into bile salts. This demonstrates the potential direct role of LBP in regulating blood cholesterol levels [17].

Nonalcoholic fatty liver disease is an increasing problem in Asia, with a recent cross-sectional study on 2493 Chinese healthy volunteers showing that 42% of subjects already having clinical disease, a figure that is significantly higher than that of other populations [18]. In an animal model of nonalcoholic steatohepatitis (NASH), rats were fed a high fat diet for 12 consecutive weeks to induce disease and divided into three groups. The first group was given LBP concurrently throughout the 12-week course, and the second group was given LBP late in the study period. The third group was not given LBP throughout the study period. The rats that were given LBP throughout the 12-week course of high fat diet demonstrated no significant differences in terms of hepatic cellular architecture compared to negative controls [19]. For rats that were given late LBP treatment from 9 to 12 weeks, although there was positive histological evidence of steatohepatitis, there was a marked reduction in lipid droplet accumulation and reduction in hepatocyte necrosis compared to livers of untreated rats [19]. Sirius red staining also demonstrated reduced collagen secretion and thus attenuated hepatic fibrosis, in both LBP treatment groups compared to untreated rats. Additionally, it was also established that giving 1mg/kg LBP from the 3^rd^ to 12^th^ week to NASH inflicted rats significantly reduced mean body weight in treated rats compared to that in untreated rats (340.2 ± 13.4 g versus 401.7 ± 10.7 g). Significant weight reduction was also noted in NASH rats commencing LBP treatment at the 9^th^ to 12^th^ week (352.1 ± 14.0 g) compared to the untreated NASH rats (p<0.05) but this reduction is notably less than rats with early LBP treatment. [19]. Proinflammatory markers such as tumor necrosis factor alpha (TNF-*α*), IL-1*β* a, cyclooxygenase (COX-2) and Monocyte Chemoattractant Protein-1(MCP-1), and profibrotic factors, including TGF-*β*1, *α*-SMA, and Smad2/4 expression were significantly raised in NASH rats, but this rise was either eliminated or attenuated significantly in both LBP-treated groups. In an in vitro model of NASH on a rat hepatocyte cell line using sodium palmitate (SP), LBP treatment was compared to l-arabinose and *β*-carotene treatment. While the results showed that all three treatments were capable of abolishing hepatic fibrosis, LBP was demonstrated to have the strongest effect [19]. Similarly, in a mouse model of NASH, mice were first fed a fat rich diet for 2 months and then orally given varying concentrations of LBPs once every day for 2 months. The results showed that mice fed with high fat diet had significantly lower levels of hepatic antioxidant enzymes such as superoxide dismutase (SOD), glutathione peroxidase (GPx), and catalase (CAT) with lower levels of GSH than negative controls. With LBP treatment, there was a significant dose-dependent rise in antioxidant enzymes [20].

The effect of LBP in ameliorating hepatic damage extends to situations where the insult is direct toxicity. In an animal model of hepatic fibrosis, rats were given carbon tetrachloride (CCL4) for disease induction and then given different concentrations of LBP powder as treatment. Results showed a clear negative dose-response relationship between LBP powder concentration given and blood levels of the hepatic enzymes ALT and AST. The paper further showed the ability of LBP to decrease hepatic SOD and GSH-Px activity while simultaneously inhibiting CCl4-induced up-regulation of TNF-*α*, IL-1*β*, and MCP-1 mRNA levels [21].

#### 3.1.2. Neuroprotective Effects

LBP's neuroprotective effects are one of the most widely studied fields regarding LBP's therapeutic effects. According to the World Health Organization, stroke is the 2^nd^ most common cause of death only behind ischemic heart disease. Those that do survive an episode of stroke are often left with a significant degree of lifelong disability. Overall, from animal models, LBP is a safe substance that has the potential to improve outcomes in patients with stroke. The optimal dosage and timing of administration however remains to be investigated in clinical trials.

Yu et al. used an in vitro model to study the effects of LBP in oxygen glucose deprived/reperfused (OGD/R) primary hippocampal mice neuron cells [22]. LBPs of 15ug/ml, 30ug/ml, and 60ug/ml concentrations were used in the pretreatment of the hippocampal neuronal cells. They found that LBP significantly increased cell viability, reduced lactate dehydrogenase (LDH) levels, and reduced reactive oxygen species in a dose-dependent manner. Apoptotic proteins such as cleaved Caspase-3, Caspase-3, Bcl-2, and Bax were also evaluated using Western blot, showing upregulation of Bcl-2/Bax protein ratio and downregulation of the ratio of cleaved Caspase-3/Caspase-3 in LBP treated groups. LBP treatment was also shown to reduce Beclin 1 and LC3II/LC3I levels while increasing p62 expression, thereby promoting antiautophagic effects [22]. It was further demonstrated that the observed neuroprotective effects of LBP in this experiment were mediated through the PI3K/Akt/mTOR signaling pathway. [22].

Liu et al. performed a study on the effect of LBP on streptozotocin-induced diabetes mellitus Sprague-Dawley rats subjected to middle cerebral artery occlusion (MCAO) ischemia reperfusion brain injury [23]. There were 4 different animal groups, i.e., the normoglycemic group (NG), hyperglycemic group (HG), LBP treatment group (intraperitoneal LBP injection at 25mg/kg/day for 4 weeks prior to surgery), and an insulin treatment group (2 U/day). Within each group, the rats were divided into receiving sham surgery, MCAO with 24 hours of reperfusion and MCAO with 72 hours of reperfusion. [23]. Upon assessment, it was shown that LBP treated rats had significantly reduced cerebral infarct volumes 24 hours after reperfusion and histopathological analysis demonstrated that LBP treated rats had significantly less pyknotic cells 24 hours and 72 hours after reperfusion. Neurological defect scores and T-maze test times demonstrated that LBP pretreated and insulin-treated rats had improved neurological recovery compared to hyperglycemic controls [23].

LBP has also been shown to improve memory and neurogenesis. This in turn could be of benefit to patients suffering from Alzheimer's disease and other forms of dementia. Scopolamine (SCO) is an acetylcholine receptor (mAChR) antagonist, which impedes memory and learning similarly to what is observed in Alzheimer's disease patients [24]. Chen et al. conducted experiments on scopolamine-treated Sprague Dawley rats. The animals were divided into a vehicle/saline (veh/sal) group, vehicle/SCO (veh/SCO) group, and LBP/SCO group (LBP either 0.2 mg/kg or 1mg/kg per day) [4]. Behavioral tests, including the Novel Object Recognition (NOR) test and Object Location Recognition (OLR) test, found that LBP-treated rats used more time exploring the novel objects with increased discrimination index compared to scopolamine-treated controls [4]. Immunofluorescence for cell proliferation in the hippocampus showed a significantly higher number of Ki67-immunoreactive nuclei in LBP/SCO group (165.0±30.7) compared to veh/SCO group (52.0±19.4) [4]. Furthermore, immunohistochemistry for DCX-immunoreactive neurons revealed that LBP treatment raised DCX-positive neurons to 685.5±132.6 DCX-positive per field from 25.4±15.2 DCX-positive cells per field in the SCO treated group. The observed neuroprotective effect was shown to be independent of competition for acetylcholine receptors between LBP and scopolamine [4].

The neuroprotective effects of LBP have also been demonstrated to extend to the retina of the eye as well. Yang et al. used C57BL/6 male mice to induce transient retinal ischemia by blocking the internal carotid artery and mice were fed LBP at 1 or 10mg/kg or the vehicle (phosphate buffered saline) with a gastric tube for 7 days [25]. The mice were divided into Groups A and B. Group A did not receive retinal ischemia and was fed 7 days with LBP. Group B received the transient retinal ischemia and continued LBP treatment 1, 3, or 7 days after retinal ischemia. Electroretinogram was used to study the retinal function and showed that there was a significant reduction in the b-wave and oscillatory potentials (OP) amplitude in vehicle treated mice compared to sham surgery (P<0.001) [25]. On the contrary, mice given 10mg/kg LBP treatment had significantly preserved b-waves and OP amplitudes (P<0.05). Upon examination of the retinal morphology, the ganglion cell layer (GCL) and inner nuclear layer (INL) cells were obviously lost in vehicle-treated mice compared to sham surgery. With LBP treatment the cellular organization of the retinal layers remained relatively normal with less pyknotic nuclei with significantly increased viable GCL cells in 10mg/kg LBP treatment group compared to control (P<0.05) [25]. Bipolar rod cells were studied via Protein Kinase C-*α* (PKC-*α*) immunostaining. PKC-*α* immunoreactive cells were significantly reduced in vehicle treated cells compared to sham (p<0.05) but LBP treated retinae showed increase PKC-*α* immunoreactive cells [25]. Glial response was evaluated with glial fibrillary acidic protein (GFAP) and glutamine synthetase (GS) staining. There was significantly stronger staining of GFAP in vehicle-treated compared to control but with LBP treatment the GFAP staining was notably reduced suggesting less gliosis when giving LBP treatment [25].This study demonstrates LBP's ability to protect retinal neurons and could be applied in the prevention or slowing the progression of diseases such as diabetic retinopathy, glaucoma, and retinopathy of prematurity.

#### 3.1.3. Antiaging Effects

The antioxidant effects of LBP have been studied in a number of animal models and cell culture experiments. One in vitro study demonstrated LBP's capabilities in clearing superoxide, hydroxyl radicals, and 2,2-diphenyl-1-picrylhydrazyl (DPPH) free radicals, with clearance levels increasing with LBP concentration and levelling off at 47.158 ± 6.231 *μ*g/ml [26]. Another study investigated LBP's antioxidizing effects in Wistar mice using the D-galactose (D-gal) aging model [27]. D-galactose has been linked to cognitive and motor skill deterioration typically seen in aging [28]. The low dose group received a subcutaneous injection of 100mg/kg/day D-gal and gastric infusion of 10ml/100g/day LBP solution for 30 days. The medium and high dose group was given 20ml/100g/day and 40ml/100g/day LBP solution, respectively, in combination with 100mg/kg/day of D-gal. These groups were compared to an only D-gal treated group and controls. The serum and skin on the back of the mice were collected for sampling. In the only D-galactose group, the levels of superoxide dismutase (SOD), catalase (CAT), and glutathione peroxidase (GSH-px) were significantly reduced compared to controls (P<0.01). For LBP and D-gal treated groups, the levels of SOD and hydroxyproline (Hyp) in the skin were comparatively raised significantly in a dose-dependent manner [27]. SOD is important in free radical scavenging. Its downregulation leads to premature aging of the skin, with features such as loss of collagen and skin wrinkles [29]. As a result, there has been a rise of LBP based face creams available in the market, although its effects have yet to be confirmed in human clinical studies.

In zebrafish, it was shown that giving LBP reduced the expression of proaging genes such as p53, p21, and Bax, while increasing expression of telomerase reverse transcriptase [30]. The therapeutic effect was demonstrated at LBP concentrations of 3.0 mg/ml. Using acridine orange staining to assess the antiapoptotic effect of LBP, it was shown that LBP concentrations from 1 to 3 mg/ml demonstrated dose-dependent antiapoptotic effect with apoptosis induction at 4mg/ml. Senescence associated-*β*-galactosidase (SA-*β*-gal) staining at pH 6.0 was also performed and it was found that staining at 1, 2, and 3 mg/ml LBPs was 88.3%, 81.7%, and 68.3%, respectively of the staining in the control. This is important as SA-*β*-gal is known to be an in vivo and in vitro senescence marker [31].

#### 3.1.4. Anticancer Effects

*(a) Breast Cancer.* LBP also plays an important role in activating extracellular signal-regulated kinase 1/2 (Erk1/2), which in turn affects p53 expression in a dose-dependent manner [32]. Moreover, there was a significant negative correlation between the LBP treatment concentration and the distribution of cells in the G0/G1 phase from 49.06% to 22.68% with a significant increase in the number of LBP-treated cells arrested in S phase from 45.29% to 71.10%. This demonstrates LBP's tendency to inhibit cancer cell proliferation in vitro. Similar effects were also demonstrated in colorectal cancer cell lines SW480 and Caco-2 cells, with concentration of LBP negatively affecting cell proliferation [33] and cell adhesion [33]. A potential mechanism by which LBPs exert its anticancer effects is by regulating tumor apoptosis via the Bax and Bcl-2 expression and induce cell cycle arrest of a variety of cancer cells at G0/G1, S, or G2/M phase [6].

*(b) Colon Cancer.* A murine colon cancer cell line, CT26-WT, was treated with different concentrations of LBP (0 *μ*g/ml, 1 *μ*g/ml, 10 *μ*g/ml, and 100 *μ*g/ml) [34]. Mice bone marrow was also collected and incubated in the necessary solutions for dendritic cell collection. LBP treatment-initiated Notch signaling in dendritic cells was found. Specifically, mRNA expressions of Notch, Jagged, Hes1, and Hes5 were raised with LBP treatment. This is significant as Notch signaling is a key initiation process in dendritic cell differentiation, as the notch ligands serve as binding domains. Moreover, LBP treatment also significantly increased cytotoxicity of dendritic cell-meditated cytotoxic T-lymphocytes on the CT26-WT colorectal cancer cells. This was demonstrated by the presence of a significantly greater proportion of CD3+CD8+T cells after 4 days of treatment with LBP compared to controls (80.9±7.93% vs 54.5 ±4.26%) [34]. Thus, apart from cell cycle regulation, LBPs also has the ability to use immunoregulation to target tumor cells.

*(c) Cervical Cancer.* Zhu et al. investigated the effects of different concentrations of LBP solution (0, 3.125, 6.25, 12.5, 25, 50, 100, and 200 mg/L) on human cervical cancer HeLa cells. Using the MTT assay to assess cell proliferation, it was found that a concentration of 6.25 mg/L LBP solution exerted the greatest cytotoxic effect after 4 days of treatment, with the percentage of cell inhibition being 35% (P < 0.05) [35]. Moreover, LBP also affected cell cycle progression in proliferating HeLa cells. It was found that culturing with LBP medium led to significant accumulation of cells arrested in S phase, with cells in G0/G1 phase significantly being reduced from 56.8% to 31.4% [35]. Consistent with the effect observed in breast cancer cell lines, LBP appears to promote apoptosis of cancerous cells by preventing cell cycle progression. The use of nontoxic agents such as LBP to treat cancer would tremendously impact the quality of life of patients fighting cancer.

#### 3.1.5. Immunomodulatory Effects

The effect of LBP on B and T cell activation was investigated using a mouse model with female BALB/c mice. There were 4 groups (with n=5 per group) being injected with either LBP alone or LBP + rAd5VP1 in 0.2 ml of PBS. Negative control groups were being injected with PBS + rAd5VP1. The BALB/c mice were then given 5 mg/kg, 25 mg/kg, or 50 mg/kg of LBP perorally for 7 days. Helper T cells were characterized by CXCR5 and PD-1 surface markers. For 25mg/kg and 50mg/kg LBP-treated groups, the PD1+CXCR5+ T cells percentage was significantly raised at 3.93 ± 0.74% and 3.84 ± 0.20%, respectively [36]. It is also worth noting that the IL-21 measured in 25mg/kg 50mg/kg LBP-treated groups were significantly higher compared to controls. IL-21 plays a key role in helper T cell differentiation and B cell activation [36]. To evaluate the germinal centers of B cells, the spleen of the mice was harvested and flow cytometry was used to quantify B220+GL-7+ germinal center B cells. Upon treatment with either 25 mg/kg or 50 mg/kg of LBP solution, there was significantly higher proportion of splenic B220+GL-7+ B cells on flow cytometry compared to controls [36]. This study showed that part of the immunomodulatory effects of LBP is through attenuating humoral immunity through helper T cells [36].

In a similar study, BALB/c mice were injected with nanoliposome encapsulated LBPs in ovalbumin (LBPL-OVA) for 2 weeks compared to the control [37]. Apart from LBPs effect on immunity, the effect of LBP encapsulated in nanoliposomes was compared to that without encapsulation (LBP-OVA). The positive control group was injected with Complete Freund's Adjuvant and OVA (CFA-OVA) [37]. Antigen transport to the draining lymph nodes was assessed via immunohistochemistry. It was demonstrated that LBPL-OVA-injected mice had significantly higher levels of antigen concentration after 1 week of injection compared to other groups. It was also shown that LBPL-OVA mice had significantly increased CD4+ and CD8+ T cells compared to CFA-OVA groups, with CD3+ T cells significantly raised compared to other groups [37]. Thus apart from improving humoral immunity, LBPs can also induce cell-mediated immunity and, by improving antigen delivery to the lymph nodes (the key site in initiating adaptive immunity), it can facilitate T cell and B cell activation by antigen presentation by antigen presenting cells [37].

### 3.2. Local or Topical Effects

#### 3.2.1. Effects on Cornea

Du et al. evaluated the effect of LBP rat corneal epithelial cells after UV-B light damage. The experiment demonstrated LBPs ability to increase cell viability and reduce apoptosis after UV-B light damage. The optimum concentration of LBP, as reported by the study, was 1mg/ml [38]. Quantitative PCR demonstrated significant upregulation of Bax mRNA by approximately 2-fold and downregulation of Bcl-2 mRNA by approximately 0.27-fold with around 8-fold increase in the Bax/Bcl-2 ratio in UV-B treated rat corneal epithelial cells compared to those treated with sham radiation. This effect was significantly dampened when the UV-B treated rats were concurrently treated with LBP [38]. It was also shown, via western blot, that UV-B treated cornea epithelial cells had increased p-JNK (phosphorylated JNK). This in turn was inhibited through concurrent treatment with 1 mg/ml LBP solution [38]. It is thus suggested that LBP prevents apoptosis of injured corneal epithelial cells via inhibition of the JNK-Bax-caspase 3 pathway.

Chien et al. investigated the potential therapeutic effects of LBP extract on an experimental model of dry eye in rats. Tear film metrics, including tear film break-up time, Schirmer's test, and keratoconjunctival fluorescein staining, were compared between rats randomly assigned to control group, low-dose goji berry extract group (250 mg/kg/body weight), median-dose group (350 mg/kg), and high-dose group (500 mg/kg) after experimental dry eye induction [39]. LBP-treated rats had significantly higher Schirmer's test score and tear break-up time with reduced fluorescein staining compared to other groups, with a clear dose-response relationship. This shows that goji berry extract increases tear production and reduces tear evaporation, making it a potentially effective supplement to treat dry eye disease [39].

## 4. Discussion

Given its favourable safety profile, LBP has the potential to serve as an adjunctive measure in existing treatments with few, if any, concerns regarding toxicity or adverse reactions. From published studies, the therapeutic use of LBP appears to be particularly promising in metabolic diseases, including diabetes, hypercholesterolemia, and fatty liver disease. There is currently a lack of pharmaceutical agents directly treating fatty liver disease, with affected patients advised on dietary and lifestyle modification during early stages of the disease. Moreover, the promising hypoglycemic effects of LBP in diabetic patients, particularly those on dietary measures alone, suggest that it may be a suitable oral hypoglycemic supplement during the early phase. Regarding the use of LBP in cancer treatment, however, the evidence is predominantly in vitro, where high concentrations of LBP are required for significant cytotoxic effects. In practice, it would be questionable whether LBP supplementation would provide any additional benefit in the clinical outcome of cancer patients at nontoxic concentrations. In terms of its immunomodulatory properties, LBP has shown positive effects in boosting both humoral and cell-mediated immunity quantitatively and qualitatively. It would be worthwhile to assess LBPs immune cell-boosting effects using disease models of acquired immunodeficiency, such as HIV.

As a topical agent, the use of LBP in the management of ocular surface disease is very promising. Dry eye disease is common in our population with significant impact on quality of life [40]. Given the cornea's superficial and easily accessible location, we could also consider the use of contact lenses as a drug delivery system for LBP. The use of latanoprost-eluting contact lenses has already shown promising results in combating poor long-term drug compliance in glaucomatous optic neuropathy [41]. Furthermore, scleral contact lens systems have been used as a method to effectively deliver bevacizumab to the ocular surface as a method to treat sight-threatening corneal neovascularization [42].

When comparing LBP to another popular supplement, lutein, the latter is much more readily available in pharmacies and convenience stores. Lutein also has substantial evidence for its protective effects in the eye, preventing diabetic retinopathy [43], experimental cataract formation [44], disease progression in age-related macular degeneration [45], and experimental uveitis [46] in published studies. Like LBP, lutein is a well-established antioxidant but unlike LBP lacks evidence of systemic benefits such as in glycemic control and immunomodulation.

One major concern in the interpretation of published studies on the subject is the variation in source of LBP used. Unlike lutein, there is no research-grade LBP currently available in the market. Thus, for experiments, researchers must either extract their own LBP from wolfberries or, more commonly, source from a number of Chinese medicinal companies producing LBP powder for consumption use. At the moment a wide range of concentrations of LBP is available in the market, with a lack of details on extraction and purification processes used. The lack of a standardized source for LBP and lack of quality control means that the results obtained from experiments will inevitably be affected by the source of LBP used by the research team. Introducing methods to objectively quantify LBP, through methods such as high-performance liquid chromatography, will allow a better comparison between experiments. 

*Future Directions.* One of the key areas for further research is to develop a viable method of drug delivery to achieve therapeutic levels in the form of topical treatment such as LBP eye drops and systemic formulas such as LBP powder capsules, LBP containing injections, or oral intake of wolfberries each day and whether they should be consumed in its natural form or as a tonic/broth and the respective doses needed. This is a concern as we have yet to understand whether LBP is heat stable and if we use the traditional boiling of wolfberries to form tonics/broths we may compromise their active ingredients. In addition, we should also assess the bioavailability of each route of administration and their safety profile.

## 5. Conclusion

LBPs have a number of promising therapeutic uses in the liver disease, metabolic disorders, immune dysfunction, aging, neuroprotection, cancer, and ocular surface diseases. These potential applications should be thoroughly assessed through further in vivo studies and ultimately in well-designed randomized controlled clinical trials. One major limitation of current research is a lack of standardization and quality control for the LBP used. The availability of research-grade LBP, of consistent concentration, will help promote further interest and robust research work in this field.

## Figures and Tables

**Table 1:** Clinical studies demonstrating the therapeutic effects of LBP.

Source	Country	Groups^a^	Sample size	Method^b^	Parameters^c^	Outcomes^a,d^	Remarks^d^
Cai et al., 2015 [13]	China	Type II DM subjects: LBP 300mg/day vs Type II DM control	37/30	Double blind RCT	Serum glucose	Glucose AUC decreased in LBP group vs placebo (-7.86 % vs. 1.61 %)	RCT evidence for clinical efficacy of supplementary LBP treatment for vascular risk factor control in type II DM subjects However, potential impact limited by a small sample size
					Insulinogenic index	Increased from -0.98 % to 0.04 % in LBP treated	
					Serum lipid	Significantly raised HDL in LBP compared to control	

Amagase and Nance, 2011 [16]	United states	Overweight subjects: LB juice (30/60/120ml) vs control	8	Double blind, placebo controlled RCT	RMR + PPEE	LB group: 58.26±5.72ml/min (VO2) increase 1hr after intake, significantly higher than baseline	RCT evidence for clinical efficacy of supplementary LBP treatment in waist circumference reduction for overweight subjects Mechanism suggested to be through increase in basal metabolic rate However, it is important to note that change in VO2 is a crude measurement for basal metabolic rate
			14/19		Waist circumference	LB group: 5.54±0.65cm reduction after 15 days treatment compared to baseline	

*Total papers = 2*							

*Abbreviations.* a-Lycium barbarum, b-randomised control trial (RCT), c- resting metabolic rate (RMR), postprandial energy expenditure (PPEE), d-area under curve (AUC), high-density lipoproteins (HDL), and breath oxygen volume (VO2).

**Table 2:** Translational studies demonstrating the systemic therapeutic effects of LBP.

Source	Country	Groups^a^	Sample size	Method	Parameter(s)^b^	Outcomes^c,a^	Remarks
*A. Metabolic effects*							
Pai et al. 2013 [15]	India	Mice: ND/HFD/HFD +ATV/HFD +LB (10mg/kg/day) /HFD +LB (20mg/kg/day)	6/6/6/6/6	In vivo	Blood lipid profile	TC, TG, VLDL: significant reduction in both LBP groups	Demonstrates improvement in lipid profile in LABP supplementation groups
						HDL: significant increase in LB 250mg/day only	
						LDL: significant reduction in LB 500mg/day	

Zhu et al, 2015 [17]	China	Normal control group (NC) vs hyperlipidemia group (H) vs hyperlipidemia + LBP group (HL) vs hyperlipidemia + chronic composite psychological stress group (HS) vs hyperlipidemia + LBP+ chronic composite psychological stress group (HLS)	9/9/9/9/9	In vivo	Blood lipid profile	H group had significantly higher TG and TC than NC group (P<0.05 & P<0.01). HL group had significantly lower TC and TG (P<0.05 for both) compared to H group. HLS group had significantly less TC and TG than NC and H group (P<0.01 and P<0.05 respectively).	Interesting study highlighting the possible molecular mechanism as to how LBP works which could be applicable to other disease models
					Hepatic MDA levels	MDA higher in each of the H and HS groups, as compared with the NC group (P<0.05 and P<0.01, respectively).	
						Hepatic MDA in HL and HLS groups were lower relative to H group (P<0.05)	
					ELISA for HSP-70	Increase in HSP-70 were observed in the HL and HLS groups (P<0.05 and P<0.01) while HSP-70 significantly lower in the HS group compared to H group (P<0.05)	
					ELISA for Il-6	HS group had increased IL-6 which was reduced with LBP treatment (P<0.05 compared to HS group and P<0.01 compared to H group)	
					Reverse transcriptase- quantitative polymerase chain reaction	mRNA CYP7A1 increased in the HL group compared with H and HS groups (P<0.05). CYP7A1 significantly reduced in the HS group (P<0.01)	

*B. Anti-aging*							
Zhang et al., 2013 [26]	China	LBP effect on free radical and ROS clearance rate	None	Chemical study	OH- clearance rate	Up to 89.45% clearance and IC50: 6.45*μ*g/ml	Results of limited value as no cells or animals were involved in this study. Results can be basis for in vivo trials
					Superoxide clearance rate	IC50 is 7.13*μ*g/ml	
					ABTS clearance rate	IC50 47.158±6.231ug/ml	

Liu & Yi et al, 2013 [27]	China	Rats: Control vs 100 mg/kg/day D-gal + 1 ml/100g/day saline or 10 ml/100g/day LBP or 20 ml/100g/day LBP or 40 ml/100g/day LBP or 5mg/100g	10/10/10/10/10/10	In vivo	SOD, MDA, CAT and GSH-px in mouse blood and mouse skin	Significant increase in MDA and reduced SOD, GSH-px and CAT of aging group compared to normal group (p<0.01)	Only assessed the levels of reactive oxygen species and free radicals but not the clinical implications of the effect. The effect of LBP on the skin could have been further investigated such as collagen organization
						LBP increases SOD, GSH-px and CAT significantly and reduces MDA in the aging group (p<0.01)	

Xia et al., 2014 [30]	China	Zebrafish embryos: Control vs LBP of 1, 2, 3 or 4 mg/ml for 3 days	30	In vivo	Senescence assessment	AO staining: cell apoptosis reduced in 1–3 mg/ml LBPs in a dose-dependent manner,4mg/ml LBPs induced apoptosis	Demonstrates dose-dependent anti-apoptotic effects of LBP in vivo
						SA-*β*-gal quantification: 1, 2 and 3 mg/ml LBPs was 88.3%, 81.7% and 68.3% respectively of the staining observed in the control	
					P53 signaling pathway gene expression	p53, p21 and Bax decreased in LBP treated compared to control	

*C. Hepatic effects*							
Xiao et al., 2014.[19]	China	Rats with NASH: Control vs LBP (0-12 weeks) vs LBP (9-12 weeks) vs NASH vs NASH +LBP (12 week) vs NASH + LBP (9-12 week)	6/6/6/6/6/6	In vivo and in vitro	Serum ALT, TNF-*α*, IL-1*β*a, COX-2 and MCP-1 levels	Reduced in LBP + NASH groups compared to NASH alone	Demonstrates beneficial effect of LBP supplementation directly through reduction in hepatitis and indirectly through reduction in body weight and insulin resistance
					Body weight	340.2 ± 13.4g (LBP 3-12 weeks + NASH) vs 352.1 ± 14.0g (LBP 9-12 weeks + NASH) vs 401.7 ± 10.7g (NASH)	
					Insulin resistance	Significantly reduced in LBP treated NASH rats compared to n treatment	

Gan et al. 2018 [21]	China	Wistar Rats: Control vs CCl4 vs CCl4 + LBP (400 mg/kg, 800 mg/kg and 1600 mg/kg)	10/10/10/10/10	In vivo	ALT(U/L)	45.64 ± 3.49/124. 8 ± 9.78/ 89.69 ± 5.36/64.58 ± 4.95/ 60.12 ± 4.46c	Demonstrates anti-oxidant and anti-inflammatory effects of LBP supplementation in an in vivo model of hepatic injury
					Anti-oxidizing enzymes	LBP reversed decreased SOD, GSH-Px activities, GSH levels & reversed increases of MDA levels	
					Pro-inflammatory cytokines	CCl4 + LBP 400 mg/kg, 800 mg/kg & 1600 mg/kg inhibited mRNA of TNF-*α*, IL-1*β* & MCP-1	

*D. Anti-cancer effects*							
Mao et al., 2011 [33]	China	MCF-7 cells treated with 0, 10,30, 100, or 300 *μ*g/ml LBP for 24h	None	In vitro	Cell cycle distribution	G0/G1 phase: 49.06% to 22.68%). S phase: 45.29% to 71.10%	Demonstrates potential cytotoxic effects of LBP through inhibition of cell proliferation. This effect may not be limited to cancer cells.
					P53 and P21 expression	LBP decreased p53 and p-p53 and p21	

Zhu and Zhang, 2013 [35]	China	HeLa cells, a human cervical carcinoma cell line treated with LBP (0, 6.25, 25 or 100 mg/ x4 days)	None	In vitro	HeLa cell proliferation	4 days LBP treatment at 6.25 mg/ml showed greatest inhibition of 35%	
					cell cycle distribution	G0/G1 phase decrease significantly from 56.8% to 31.4% with LBP treatment	
						Accumulation of cells in the S phase (33.5–59.4%) with LBP treatment	

Wang et al. 2018 [34]	China	CT26-WT murine colon cancer line: LBP (0 *μ*g/ml, 1 *μ*g/ml, 10 *μ*g/ml, and 100 *μ*g/ml for 24/48 hours) cytotoxicity on	None	In vitro	LBP effect on DC-mediated CTL cytotoxicity	Proportion of CD3+CD8+ cells increased LBP for 4 days compared to control (80.9±7093% vs 54.5±4.26 %)	Demonstrates potential anti-cancer effects of LBP through priming of cell-mediated immunity
					Notch signaling in dendritic cells	Increased expression of Notch, Jagged, Hes1 and Hes5 upon LBP treatment	

*E. Immunomodulatory effects*							
Bo et al., 2017 [37]	China	Mice: Control vs CFA-OVA vs OVA vs LBP-OVA vs BL-OVA vs LBPL-OVA	20/20/20/20/20/20	In vivo	CD3+ and CD4+/CD8+ T cell activation	All increased in LBPL-OVA injected mice	Demonstrates beneficial effects of LBP supplementation on cell-mediated immunity
					Antigen transport to LN		

Su et al., 2014 [36]	China	Mice: Control vs LBP 5/25/50 mg/kg for 7 days per os after immunized with LBP or LBP + rAd5VP1	5/5/5/5	In vivo	Follicular helper T cell generation	PD1+CXCR5+ Tfh cells: 5 mg/kg (2.17 ± 0.07%) 25 mg/kg (3.93 ± 0.74%), 50 mg/kg (3.84 ± 0.20%)	Demonstrates potential beneficial effects of LBP on cell mediated and humoral immunity
					Germinal center formation	B220+GL-7+ B cells: Control (1.80 ±0.49%),5mg/kg ((2.68 ±0.09%), 25mg/kg (3.95 ± 0.51%), 50mg/kg (4.00± 0.41%)	

*F. Neuroprotective effects*							
Chen at al, 2014 [4]	China	Rats: vehicle/saline vs vehicle/SCO vs LBP/SCO (LBP either 0.2mg/kg/day or 1mg/kg/day)	12/10/11	In vivo	NOR and OLR Task	Vehicle/SCO DI: 51.4±7.5% LBP/SCO DI: 65.6±18.6%	Well conducted study showing LBP's protective effect in the hippocampus whole also highlighting its limitations.
					Morris Watermaze	LBP/SCO significantly decreases in the latency time and swim distance compared to SCO-treated	
					Ki67 immunostaining	Ki67-immunoreactive nuclei were significantly increased in the LBP/SCO group (165.0±30.7) versus the vehicle/SCO group (52.0±19.4)	
					IHC for DCX-Positive neurons	Vehicle/saline: 566.2±112.3 DCX-positive cells per field vs Vehicle/SCO:25.4±15.2 DCX-positive cells per field vs SCO/LBP: 685.5±132.6 DCX-positive cells per field	
					AChE in hippocampus	AChE was significantly raised in SCO-treated compared to control, but LBP treatment resulted in no significant reduction.	

Yu et al., 2018 [22]	China	Primary hippocampal neurons from C57BL/6 mice embryos	None	In vitro	MTT assay	OGD/R group had significant reduction of cell viability (p<0.01) with dose dependent increase of viability with LBP treatment	Thorough study which not only showed LBP's neuroprotective effects but also the underlying mechanism and a good basis in studying the effect of LBP on Alzheimer's disease in clinical trials. There is still a matter of whether LBP can prevent the onset of neurodegenerative diseases or mainly slow the progression once they've occurred.
					Lactate dehydrogenase (LDH) leakage assay	Dose-dependent reduction in LDH release with LBP pretreatment	
					Western blot for apoptotic proteins	Bcl-2/Bax protein ratio was up-regulated ratio of cleaved Caspase-3/Caspase-3 was down-regulated in LBP treatment groups	
					Western blot for autophagic proteins	Beclin 1 expression and LC3II/LC3I ratio were reduced, p62 expression increased in LBP pretreatment	
					PI3K/Akt/mTOR signaling pathway analysis with Western blot	PI3K-specific inhibitor (LY294002) increased the expression of LC3II and cleaved Caspase-3 compared to LBP 60ug/ml	

Liu et al, 2017 [23]	China	Rats: NG MCAO vs HG MCAO vs HG MCAO with LBP vs HG MCAO with insulin	27/29/29/29	In vivo	Infarct volume assessment	HG group had infarct volume than NG (p<0.05) while LBP and insulin groups had significantly reduced infarct volumes 24 hours after reperfusion (p <0.05)	Promising results regarding LBP's effect on stroke which carries high mortality and morbidity globally. Results are a good basis for clinical trials.
					Neurological deficits score	HG group had more severe neurological deficit scores than NG group (p<0.05) while LBP or insulin treatment reduced the deficit score at 24 & 72 hours of reperfusion (p<0.05)	
					T-maze	HG group spent less time than the NG group at target arm (p<0.05) while LBP or insulin groups spent longer times at target arm than the HG group at 24& 72 hours of reperfusion (p<0.05).	
					H&E staining	HG group had significantly more pyknotic nuclei than NG group (p<0.05) while LBP or insulin pre-treatment reduced neuronal pyknosis at 24 and 72 hours reperfusion (p<0.05).	
					Western blot	Opa1 significantly increased in LBP group compared to HG group at 24 hours reperfusion while ratio of phospho-Drp1/Drp1 was decreased in LBP group at 24&72 hours reperfusion (p<0.05)	

Yang et al, 2017 [25]	Hong Kong	Mice: Group A- LBP treatment without retinal ischemia vs Group B- sham/ vehicle/ 1mg/kg or 10mg/kg of LBP	6/5/5/5/5/5	In vivo	Electroretinogram	LBP 10mg/kg treatment preserved b‐wave and OP amplitudes compared to vehicle treated (p < 0.05)	Results are a good basis for testing in patients with retinal disorders such as diabetic retinopathy and glaucoma.
					H&E stain	LBP treated had less pyknotic nuclei were noted in the GCL and INL and increased viable cells in GCL compared to vehicle‐treated group	
					PKC‐*α* immunoreactivity	LBP treated retinae had stronger PKC‐*α* immunoreactivity compared to vehicle	
					GFAP immunohistochemistry	Reduced GFAP positive staining in LBP‐treated retinae compared to vehicle-treated	

*Total papers= 16*							

*Abbreviations.* a-normal diet (ND), high fat diet (HFD), atorvastatin (AVT), *Lycium barbarum* polysaccharide (LBP), reactive oxygen species (ROS), non-alcoholic steatohepatitis (NASH), Carbon tetrachloride (CCl4), Michigan Cancer Foundation-7(MCF-7), middle cerebral artery occlusion (MCAO), normoglycemic (NG), hyperglycemic (HG), scopolamine (SCO) b- alanine transferase (ALT), tumor necrosis factor alpha (TNF-*α*), interleukin 1 beta (IL-1*β*a), cyclooxygenase 2 (COX-2), Monocyte Chemoattractant Protein-1 (MCP-1), dendritic cells (DC), cytotoxic T lymphocytes (CTL), novel object recognition (NOR), Immunohistochemistry (IHC), hematoxylin and eosin (H&E), 3-(4,5-dimethylthiazol-2-yl)-2,5-diphenyltetrazolium bromide(MTT), Glial fibrillary acidic protein (GFAP), Protein kinase C (PKC), doublecortin (DCX), Enzyme-linked immunosorbent assay (ELISA) c- superoxide dismutase (SOD), glutathione peroxidase (GSH-Px), Malondialdehyde (MDA), total cholesterol (TC), triglyceride (TG), very low density lipoproteins (VLDL), low density lipoproteins (LDL), high density lipoproteins (HDL), acridine orange (AO), Senescence associated-*β*-galactosidase (SA-*β*-gal), area under curve (AUC), mechanism of action (MOA), chemotherapy(CT), and IC50: half inhibitory concentration.

**Table 3:** Translational studies demonstrating the topical/local therapeutic effects of LBP.

Source	Country	Groups^a^	Sample size	Method	Parameter(s)^b^	Outcomes^a^	Remarks
*Cornea and Ocular Surface effects*							
Chien et al. 2018 [39]	Taiwan	Rats: Control vs GBE 250 mg/kg/body weight vs GBE 350mg/kg/bw vs GBE 500mg/kg/bw	45	In vivo	Schirmer's test	1.3±0.4 mm vs 7.4±1.8 mm vs 8.2±1.5 mm vs 9.4±0.5 mm (all at 3 weeks)	Demonstrates potential role of
					Tear break-up time	<5 s vs 7.2±2.4s vs 7.9±2.4s vs 8.8±1.2 s (all at 3 weeks)	
					KC fluorescein staining	Control mild, moderate and severe grade 37.5, 37.5, and 25.0% vs LBP treated mild, moderate and severe grade 82.5, 12.5, and 5.0% after 3 weeks treatment	

Du et al. 2017 [38]	China	Rat corneal cell line: Control vs sham vs UVB−/LBPs+ vs UVB+/LBPs− vs UVB+/LBPs+	None	In vitro	Cell viability	5mg/ml LBP increased but 10mg/ml reduced viability	Promising results but the effect on human keratocytes should be assessed
					Cell apoptosis	UVB+/LBPs−(47.06% ± 1.83%) vs UVB+/LBPs+ (13.93% ± 1.76%)	

*Total papers: 2*							

*Abbreviations.* a-Goji berry extract (GBE), body weight (bw), lycium barbarum polysaccharide (LBP), ultraviolet radiation B rays (UVB), and b-keratoconjunctival (KC).

## Data Availability

The authors agree to make all materials, data, and associated protocols promptly available to readers without undue qualifications in material transfer agreements.
